# Can Probiotics Improve the Environmental Microbiome and Resistome of Commercial Poultry Production?

**DOI:** 10.3390/ijerph10104534

**Published:** 2013-09-25

**Authors:** Adriana A. Pedroso, Anne L. Hurley-Bacon, Andrea S. Zedek, Tiffany W. Kwan, Andrea P. O. Jordan, Gloria Avellaneda, Charles L. Hofacre, Brian B. Oakley, Stephen R. Collett, John J. Maurer, Margie D. Lee

**Affiliations:** 1 Poultry Diagnostic and Research Center/Center for Food Safety, The University of Georgia, Athens, GA 30602, USA; E-Mails: adrianaped@yahoo.com.br (A.A.P.); tkwan2@uga.edu (T.W.K.); chofacre@uga.edu (C.L.H.); colletts@uga.edu (S.R.C.); jmaurer@uga.edu (J.J.M.); 2 Merial, Athens, GA 30601, USA; E-Mail: anne.hurley-bacon@merial.com; 3 Zoetis Animal Health, Madison, NJ 07932, USA; E-Mail: andrea.zedek@zoetis.com; 4 Perelman School of Medicine, University of Pennsylvania, Philadelphia, PA 19104, USA; E-Mail: andreado@upenn.edu; 5 Loehmann Animal Health International, Waterville, ME 0491, USA; E-Mail: gavellaneda@lahinternational.com; 6 Richard B. Russell Agricultural Research Center, Agricultural Research Service, South Atlantic Area, Athens, GA 30605, USA; E-Mail: brian.oakley@ars.usda.gov

**Keywords:** antibiotic, growth promoter, microbiota, litter, probiotic, prebiotic, streptogramin, integron

## Abstract

Food animal production systems have become more consolidated and integrated, producing large, concentrated animal populations and significant amounts of fecal waste. Increasing use of manure and litter as a more “natural” and affordable source of fertilizer may be contributing to contamination of fruits and vegetables with foodborne pathogens. In addition, human and animal manure have been identified as a significant source of antibiotic resistance genes thereby serving as a disseminator of resistance to soil and waterways. Therefore, identifying methods to remediate human and animal waste is critical in developing strategies to improve food safety and minimize the dissemination of antibiotic resistant bacteria. In this study, we sought to determine whether withdrawing antibiotic growth promoters or using alternatives to antibiotics would reduce the abundance of antibiotic resistance genes or prevalence of pathogens in poultry litter. Terminal restriction fragment length polymorphism (T-RFLP) paired with high throughput sequencing was used to evaluate the bacterial community composition of litter from broiler chickens that were treated with streptogramin growth-promoting antibiotics, probiotics, or prebiotics. The prevalence of resistance genes and pathogens was determined from sequencing results or PCR screens of litter community DNA. Streptogramin antibiotic usage did not elicit statistically significant differences in Shannon diversity indices or correlation coefficients among the flocks. However, T-RFLP revealed that there were inter-farm differences in the litter composition that was independent of antibiotic usage. The litter from all farms, regardless of antibiotic usage, contained streptogramin resistance genes (*vatA*, *vatB*, and *vatE*), macrolide-lincosamide-streptogramin B resistance genes (*ermA* and *ermB*), the tetracycline resistance gene *tetM* and class 1 integrons. There was inter-farm variability in the distribution of *vatA* and *vatE* with no statistically significant differences with regards to usage. Bacterial diversity was higher in litter when probiotics or prebiotics were administered to flocks but as the litter aged, diversity decreased. No statistically signficant differences were detected in the abundance of class 1 integrons where 3%–5% of the community was estimated to harbor a copy. Abundance of pathogenic *Clostridium* species increased in aging litter despite the treatment while the abundance of tetracycline-resistant coliforms was unaffected by treatment. However some treatments decreased the prevalence of *Salmonella*. These findings suggest that withdrawing antibiotics or administering alternatives to antibiotics can change the litter bacterial community and reduce the prevalence of some pathogenic bacteria, but may not immediately impact the prevalence of antibiotic resistance.

## 1. Introduction

Modern American agriculture has become successful in producing food security despite having less than 1% of the US population actually listing farming as their occupation [1]. The US Census of Agriculture estimates that 8.6 billion broiler chickens, 107 million turkeys, 96 million beef cattle, and 68 million pigs were sold for meat in 2007 with an additional 350 million laying hens and 79 million dairy cows producing eggs and milk [1]. Eighteen to 36 kg of manure are produced per day per 454 kg animal unit [2] resulting in new challenges in manure management for farmers. The United States Department of Agriculture (USDA) estimates that food animals produce 360 million tons of dry matter per year [3]. Because of increasing costs of chemical fertilizers, manure and poultry litter have become marketable as organic fertilizers. Currently the USDA allows manures to be used as fertilizer to grow organic fruits, vegetables, and grains meant for human consumption as long as specific guidelines are followed for land application and composting [4]. The US organic market has been rapidly growing, with organic food sales accounting for over 3% of total US food sales in 2008 and now it exceeds $20 billion annually in value [5]. 

Animal manure has been long known to contain varying levels of zoonotic pathogens such as *Salmonella*, *Clostridium* spp., *E. coli*, and *Campylobacter*, among others. Composting can however reduce the levels significantly [6,7], but composting of animal manures and their dissemination are much less regulated than that of human sewage biosolids [8]. The potential ecological impacts of large scale use of animal manures for land management were recently reviewed, which revealed some new concerns regarding antibiotic resistance [9]. The common perspective is that antimicrobial usage in animal production is a significant contributor to the dissemination of resistant zoonotic pathogens such as *Salmonella*, *Campylobacter* and *Staphylococcus* [10]. A number of studies have also found that manure, poultry litter, and contaminated soils contain a high abundance of antibiotic resistance genes, some of which are encoded by class 1 integrons [11,12,13,14,15,16,17,18,19]. In particular poultry litter has been shown to contain an inexplicably high abundance of resistance genes for antibiotics that are not currently used in the industry suggesting that early usage may have had an amplifying effect that has not been overcome by reducing the selective pressure for resistance [16,20,21]. 

The gastrointestinal tract is home to a diverse population of microorganisms that can have a great impact on host health. In a bird’s natural setting, gastrointestinal organisms are rapidly obtained from the egg shell and by consuming fecal material from the adult hen [22]. In today’s commercial poultry industry however, the development of a complex intestinal microbiota is delayed because, in order to reduce disease transmission, commercial hatcheries have severed the connection between the chick and the complex fecal microbiota associated with the hen. Effective application of hygiene at the hatchery allows environmental bacteria to play a larger role in seeding the intestinal tract of the commercial broiler chick [22,23]. This becomes important when you consider that the majority of the United States commercial poultry industry uses a built up litter system, meaning that the bacteria left in the litter from the previous flock(s) could potentially have a major impact, positive or negative, on the gastrointestinal integrity of the young broiler chick. Historically, commercial poultry producers have fed low levels of antibiotics in their poultry ration to negate the negative impact of pathogen growth and enteric disease on feed conversion ratio and weight gain. Virginiamycin (streptogramin A and B) is an antibiotic that has been used in agriculture for almost 20 years. Its main use has been as a growth-promoting agent in animal feed in United States and Europe and to prevent necrotic enteritis caused in chickens by *Clostridium perfringens* [24]. This antibiotic and five others were banned in the European Union in 1999 amid concern at the cross-resistances to the streptogramins used for human therapy, dalfo-/quinuprinstin [24,25]. In the US, many poultry companies are establishing antibiotic-free programs for their production system [26,27] utilizing alternatives for disease control. 

Probiotics, competitive exclusion products and prebiotics are all antibiotic alternatives that have been shown, in some studies, to prevent the establishment of pathogens in the intestinal tract of chickens, thus increasing weight gain, feed conversion ratio, and livability [27,28,29,30,31]. Probiotics have been defined as live microbial feed supplements designed to benefit the host by improving the intestinal microbial ecology [32]. Prebiotics are defined as a non-digestible food ingredient that beneficially affects the host by selectively stimulating the growth and/or activity of one or a limited number of bacteria in the intestine [33]. Prebiotics utilize complex carbohydrates that serve as nutrients for beneficial bacteria, competitive binding sites to mediate pathogen passage through the intestine, or immune modulators that reduce inflammation or stimulate the mucosal turnover rate [33,34]. 

While there have been many studies focusing on the effects of antibiotic alternatives on the microbial ecology of the poultry intestine [26,35,36,37,38,39,40,41,42,43,44,45], few studies have characterized the poultry litter microbiota by molecular ecology techniques [19,46]. The objectives of this study were to determine the microbial composition of the litter over multiple production cycles and the impact of alternatives to antibiotics on the ecology and the antibiotic resistome of broiler litter. 

## 2. Material and Methods

### 2.1. Housing, Litter and Treatments for Birds Raised on Commercial Poultry Farms

A commercial poultry company in North Georgia (USA), that uses the phosphoglycolipid antibiotic, flavomycin, as the antibiotic growth promoter (AGP) on its contract broiler chicken farms agreed to replace flavomycin with virginiamycin (streptogramin antibiotic) in the finisher feed for three successive flocks. Four houses on each of three poultry farms were enrolled in the study. The birds in two houses were administered virginiamycin for three flocks, and two houses, designated as controls, did not receive any antibiotics during the experimental period (Table 1). For houses receiving AGP, flavomycin (2.2 g/T of feed) was used in the starter, and grower feed for all flocks but only in the finisher feed for the first and fifth flocks. Virginiamycin (22 g/T of feed) was used in the finisher feed, which is provided for the last two weeks of the flocks’ growth period, for flocks 2–4. Five successive flocks on the three farms were sampled over a 12 month period. Litter samples (n = 3) from different locations in each house were collected near the end of the grow-out when the broiler chickens were seven weeks of age. 

**Table 1:** Experimental design and treatments for broiler chickens raised on 3 commercial poultry farms. Two houses on each farm received no antibiotics; two houses received flavomycin (2.2 g/T) in the starter, and grower feed and either flavomycin (2.2 g/T) or virginiamycin (22 g/T) in the finisher feed.

House	Flock 1	Flock 2	Flock 3	Flock 4	Flock 5
**A** and **B**	No AGP	No AGP	No AGP	No AGP	No AGP
**C** and **D**					
Starter-	Flavomycin	Flavomycin	Flavomycin	Flavomycin	Flavomycin
Grower-	Flavomycin	Flavomycin	Flavomycin	Flavomycin	Flavomycin
Finisher-	Flavomycin	Virginiamycin	Virginiamycin	Virginiamycin	Flavomycin

### 2.2. Housing, Litter and Treatments for Birds Raised in Research Facilities

Five 9.29 m^2^ colony houses equipped with fresh pine shaving litter were top-dressed with 75.71 L/house of built-up litter obtained from a Northeast Georgia commercial broiler house. Five hundred day-of-hatch broiler chicks were obtained from a commercial hatchery, and 100 chicks were randomly allocated to each of the colony houses at a stocking density of 930 cm^2^/bird. Birds were grown to approximately 4–5 weeks of age and received All-Lac, the combination of All-Lac and Biomos, Aviguard or Primalac; the control group did not receive any treatments. Product description and dosaging are described in Table 2. At the end of the growth period, birds were removed and the litter was left in the house for 2–3 weeks before a new flock was placed on the used litter. Each colony house was repopulated with day-old chicks from the same commercial hatchery, the designated probiotic product was applied for each treatment group again and this cycle was repeated for a total of four production cycles. Weekly, five random litter samples from each colony house were obtained using a number/grid system and pooled. A 5 g sub-sample was then taken from the pool and mixed with 20 mL of 0.9% NaCl, homogenized for two minutes and stored at −80 °C for further analysis. 

**Table 2:** Products and treatment protocols for broiler chickens raised in research facilities.

Product (Manufacturer)	Composition	Dosage and administration
All-Lac (Alltech Inc., Lexington, KY, USA)	*Lactobacillus*, *Enterococcus*, and *Pediococcus*	5 g in 600 mL water for 2,000 birds
All-Lac + BioMos (Alltech Inc., Lexington, KY, USA)	All-Lac plus a mannan oligosaccharide derived from the cell wall of *Saccharomyces cerevisiae*	All-Lac: 5 g in 600 mL water for 2,000 birds, BioMos: 2 kg/T of starter feed for 10 days, 1 kg/T of grower feed to 21 days, 0.5 kg/T of finisher feed to 35 days
Aviguard (Microbial Developments Ltd, Malvern, UK)	Undefined bacteria collected and cultured from chicken cecum contents	1 pack in 1,000 mL water for 2,000 birds
Primalac (Star-Labs, Clarksdale, MO, USA)	*Lactobacillus* species, *Enterococcus faecium*, and *Bifidobacterium thermophilum*	1 kg/T of starter to 10 days, 1 kg /T of grower to 21 days, 0.5 kg/T of finisher to 35 days

### 2.3. Quantifying Tetracycline-Resistant Coliforms in Litter and Fecal Samples

Each week, a sample was taken from the pooled sample described above and cultured to detect coliforms. In brief, 5 g was mixed with 20 mL of sterile saline solution. Samples were then serially diluted in saline and plated in triplicate onto a MacConkey agar plate and MacConkey containing 10 μg/mL tetracycline. The plates were incubated overnight at 37 °C and enumerated after 18 h by counting colony forming units (CFU). 

At the end of each cycle, 30 fecal samples were obtained from each flock. For 10 fecal samples, dilutions were made and plated as described above in order to quantify the *E. coli*. In addition, one hundred mg of each of 20 fecal samples was added to 0.9 mL of EC broth containing 10 μg/mL tetracycline in order to detect low levels of resistant *E. coli*. These were incubated at room temperature for 18 h then 10 μL plated on MacConkey agar plate containing tetracycline in order to detect low levels of resistant coliforms. 

### 2.4. Collection of the Bacterial Pellet

The protocol used for isolating bacteria from litter samples was modified from a previously published protocol [47]. Fifteen grams of each sample was suspended in 50 mL of sodium phosphate buffer (pH 8), containing 0.1% Tween 80. The suspensions were mixed vigorously for one minute and the contents poured into a filtration column consisting of prewetted crumpled gauze and cotton in a 60 mL syringe barrel placed within a 50 mL conical tube. A pellet was obtained after centrifugation at 4,500 × g for 20 min at 4 °C. The pellet was resuspended in 1 mL of sterile saline (0.9% NaCl solution in water) and centrifuged at 7,500 × g for 10 min. The resulting bacterial pellet was resuspended in sterile freezer stock medium (1% peptone, 15% glycerol in water) at a 1:10 weight:volume ratio. The samples were stored at −20 °C until processing.

### 2.5. DNA Extraction

Bacterial cells were lysed using beads, solution 1 and IRS of Mo Bio Soil DNA extraction kit (Mo Bio Laboratories Inc., Carlsbad, CA, USA) by vortexing at maximum speed for 40 min [19]. Lysed cells were treated with sodium dodecyl sulfate (final concentration, 0.5%) and proteinase K (final concentration, 0.1 μg/mL) and incubated at 37 °C for 30 min. The sample was extracted twice with an equal volume of phenol-chloroform-isoamyl alcohol (25:24:1) and once with chloroform-isoamyl alcohol (24:1). 20 µL of 10 μg/mL RNAse (DNAse-free) was added to each sample and incubated at 37 °C for 15 min. DNA was concentrated with a 0.6 volume of isopropanol and resuspended in sterile water. The presence of DNA was verified by agarose gel electrophoresis.

### 2.6. PCR Detection of Antibiotic Resistance Genes and Salmonella

DNA extracted from litter was used in PCR to detect the presence of macrolide-lincosamide-streptogramin B (*ermA-C*), streptogramin A (*vatA,B,D,E*, *vgaB*), tetracycline resistance genes (*tetM*, *tetO*) and class 1 integrase (*intI1*) and *Salmonella* as previously described [48,49]. 

*intI1* abundance was determined using quantitative real-time PCR as described by Nandi *et al*. [16]. In brief, an *E. coli* strain harboring R100-1 (GenBank accession AP000342) was used to normalize the quantitative PCR signal. The *E. coli* R100-1 strain contains one plasmid copy of *intI1* and 7 genomic copies of 16S rRNA. A standard curve relating the cycle threshold (Ct) value to gene copy number was determined for every experiment. Two replicate experiments were performed using DNA extracted from the last litter sample for each flock; replicates were acceptable if the R2 values were 0.98 or greater for both *intI1* and 16S rRNA primer sets. The number of bacteria per PCR reaction was determined by *E. coli* plate counts enabling gene copy numbers for *intI1* and 16S rRNA to be calculated from the Ct values. The number of eubacterial genomes was determined by dividing gene copy numbers by 5, the average number of 16S rRNA genes possessed by *Corynebacterium* and *Staphylococcus* species (http://rrndb.mmg.msu.edu/search.php) which are the most abundant members of the litter community. 

### 2.7. Microbial Community Structure as Defined by Terminal Restriction Fragment Length Polymorphism (T-RFLP) of 16S rRNA PCR Amplicons

T-RFLP of community 16S rRNA was used to assess changes in microflora composition as previously described [45,50,51]. Each peak’s position on a *Hae*III T-RFLP profile was matched against a Microsoft Access database of DNA fragment sizes predicted for 16S rDNA ribotypes [45] identified from a previously published litter metagene library [19], soil bacteria sequences available on the public databases [52,53] and litter sequences obtained by pyrosequencing in this study. 

### 2.8. PCR Amplification of 16S rRNA for Pyrosequencing

PCR amplification of the V3 and V6 region of the bacterial 16S rRNA genes was conducted after an initial denaturation at 95 °C for 3 min followed by 20 cycles, of denaturation at 94 °C for 30 s, annealing at 60 °C for 30 s and extension at 68 °C for 60 s. The final extension was carried out at 68 °C for 4 min. The bacterial primers (*E. coli* numbers) were 515R-NK modified and the sevenfold-degenerate primer 27F YM + 3 [54,55,56]. For ease of handling bioinformatics of pyrosequencing, the primers were synthesized with a sequencing adaptor and a specific 8-nt barcode [57]. Primers were a gift from Dr. William Whitman (University of Georgia, Athens). DNA extracted from *Salmonella enterica* Typhimurium SR11 was used as control to test for errors during PCR amplification and pyrosequencing. PCR reactions were performed on an Idaho Rapid Cycler. PCR products were visualized by electrophoresis on 1% agarose gels, stained with SYBR Green Dye (Invitrogen, Carlsbad, CA, USA) and amplicons were excised from the gel. Amplicons obtained from 3 replicates of the same samples were pooled together. Products were purified from the agarose gel initially using the Qiagen QIAquick Gel Extraction Kit, followed by the Agencourt AMpure magnetic beads (Beckman Coulter, Brea, CA, USA). Purified DNA was resuspended in water, confirmed by gel electrophoresis then concentrations were determined using a spectrophotometer. Samples were submitted to the University of Georgia Genomics Facility for pyrosequencing according to methods established by the manufacturer.

### 2.9. Statistical and Sequence Analysis

The log *E. coli* counts and gene prevalence and abundance data were analyzed using SAS. T-RFLP datasets were analyzed for similarity using the correlation coefficient function of MS Excel applied to signal intensities of the fragment patterns occurring for each sample. Shannon diversity indices were calculated using the Merlin plugin for MS Excel [58]. Analysis of 16S rRNA sequences was carried out using MOTHUR v1.21.0 [59]. DNA from *Salmonella* Typhimurium was included as a quality control for the analysis following the procedure previously established [55]. Good quality sequences were aligned using the SILVA database in MOTHUR and further filtered. 16S sequences were loaded at MG-RAST [60] in order to generate tables of genus and species frequencies at 97% similarity. Distances were calculated on preclustered sequences, and operational taxonomic units (OTU) were formed using the average neighbor method in MOTHUR. Diversity indices and rarefaction curves were calculated in MOTHUR. Libshuff was used to estimate differences between libraries composition. Statistical analyses related to the frequency of specific sequences representing microorganisms present in samples were conducted by analysis of variance using the SAS software [61]. Significant differences were based on *p* ≤ 0.05. Principal component analysis was performed using the vegan Community Ecology Package in R package version 2.0-7 (http://CRAN.R-project.org/package=vegan) [62]. To identify the prevalence of pathogenic bacteria, the FASTA sequence files deposited at MG-RAST (rast.nmpdr.org/) were compared to sequences deposited at the Ribosomal Database Project at 97% minimum similarity. The abundance of pathogens was estimated by quantifying the number of sequences exhibiting similarity to known pathogenic organisms. Statistical analysis related to the frequency of pathogens present in samples was conducted by analysis of variance using the SAS software.

## 3. Results and Discussion

### 3.1. Effects of Antibiotic Usage Changes on Microbial Community Structure of Commercial Poultry Litter

**Figure 1:**
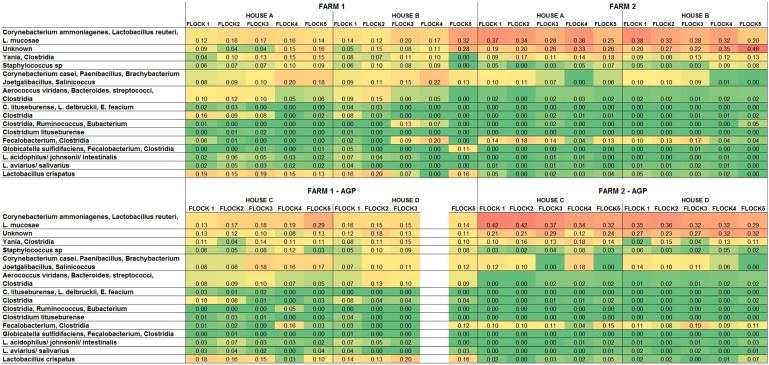
Abundance of T-RFLP peaks contributing greater than 2% of total 16S rRNA signal of litter from 5 sequential flocks of broiler chickens reared on commercial poultry farms. Two houses (C and D) on each farm contained birds that were fed antibiotic growth promoters (AGP); houses A and B were fed no antibiotics. The results are shown as the proportion of total T-RFLP signal for each flock using a green → red gradient 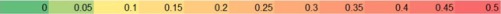.

In this study we removed antibiotic usage from two houses and changed the class of antibiotic that was used in two houses on three commercial farms; the study design is shown in Table 1, results for two farms are shown in Figure 1 and the Supplementary Material. Because of a freezer malfunction, many samples from the third farm were unavailable for testing. No treatment-associated changes were detected however inter-farm and flock differences were observed. In order to assess the composition of litter, the putative identity of each terminal fragment was inferred through *in silico* analysis of 16S rRNA sequences from a previous litter study [19] and soil bacterial sequences present in the public databases. Some species or strains of bacteria, such as *Aerococcus*, *Bacteroides*, and *Streptococcus* could not be distinguished by fragment size; therefore the compositional results are expressed as combinations of organisms. Overall results by farm and treatment are shown in Table 3 and Table 4; temporal results by farm, flock, and house are shown in Figure 1. 

There were distinct differences in the litter composition between the two farms although no apparent differences were seen associated with antibiotic usage. Farm 1 tended to have more signal predicted to represent *Aerococcus/Bacteroides/Streptococcus*, corynebacteria, *Lactobacillus*, and *Staphylococcus* while farm 2 samples produced more signal for *Corynebacterium/Lactobacillus* and unknown bacteria (Table 4). Figure 1 suggests that the litter composition displayed community successions over time with flock cycles which partially resulted from litter management practices on the farms. Fresh pine shavings are commonly applied to the top layer of the litter before each flock is placed. However there appear to be differences in the composition of the litter from the first flock to the last and some differences between houses on the same farm. 

In order to compare the composition of litter from different farms and treatments, Shannon diversity indices were used to detect whether there were differences in community structure based on the distribution of terminal fragments for each sample; correlation coefficients were used to determine similarity among the samples. Shannon diversity indices ranged from 3.27–4.11 with no significant differences noted by treatment or flock (Table 3). Richness ranged from 5–42 fragments with farm 1 mean richness (9 no-AGP, 13.75 AGP) similar to farm 2 (12.2 no-AGP, 12.5 AGP). Mean correlation coefficients were similar between houses despite treatment differences: farm 1 no-AGP 0.46, AGP 0.51; farm 2 no-AGP 0.88, AGP 0.84. When antibiotic-free houses were compared to AGP houses on the same farm, mean correlation coefficients ranged from 0.49–0.59 on farm 1 and 0.82–0.9 on farm 2. The lowest correlation coefficient (0.11) was associated with comparing the first flock cycle in one untreated house and one AGP house on farm 1, while the highest coefficient (0.98) was associated with comparing the two untreated houses for flock 1 on farm 2. In fact farm 2 correlation coefficients were consistently higher than those of farm 1 indicating significant differences among the study sites. 

### 3.2. Effects of Antibiotic Usage Changes on the Prevalence of Antibiotic Resistance Genes within the Bacterial Community of Commercial Poultry Litter

Antibiotics were not used in two houses on each farm and two houses were changed from phosphoglycolipid to a streptogramin antibiotic in the finisher feed. PCR was used to detect the presence of streptogramin-resistance genes, *ermA*, *ermB*, *ermC*, *vatA*, *vatB*, *vatD*, *vatE*, and *vgaB*, tetracycline-resistance genes, *tetM* and *tetO*, and class 1 integon, *intI*1, within litter community DNA samples. The prevalence of the genes ranged from 0%–100% but no significant difference between treatments was detected (Table 5).

**Table 3:** Diversity of bacterial communities determined by 16S rRNA T-RFLP signal of litter from 5 sequential flocks of broiler chickens raised on commercial poultry farms. Two houses on each farm contained birds that were fed the AGP flavomycin or flavomycin + virginiamycin; two houses contained birds that were not fed antibiotics.

	No antibiotic		AGP	
	Shannon Diversity Index	Richness	Shannon Diversity Index	Richness
**Flock 1**	3.94 ± 0.57	22.25 ± 4.6	4.11 ± 0.99	26.75 ± 13.1
**Flock 2**	3.68 ± 0.76	17.5 ± 8.3	3.76 ± 0.94	19.75 ± 13.3
**Flock 3**	3.85 ± 0.37	22.5 ± 6.4	3.66 ± 0.46	18.5 ± 3
**Flock 4**	3.42 ± 0.42	18 ± 5.6	3.38 ± 0.06	12.25 ± 8.73
**Flock 5**	3.27 ± 0.77	13.75 ± 6.2	3.83 ± 0.12	21.75 ± 6.8

**Table 4:** Abundance of genera contributing greater than 2% of total 16S rRNA T-RFLP signal of litter from five sequential flocks of broiler chickens raised on commercial poultry farms. Two houses on each farm contained birds that were fed the AGP flavomycin or flavomycin + virginiamycin; two houses were fed no antibiotics.

Bacterial genus predicted by T-RFLP peak	No antibiotic *		AGP *	
	Farm 1	Farm 2	Farm 1	Farm 2
*Aerococcus*, *Bacteroides*, *Streptococcus*	7.79%	1.76%	8.58%	1.45%
*Clostridia*, *Ruminococcus*, *Eubacterium*, *Fecalobacterium*	10.98%	13.70%	10.49%	11.62%
*Corynebacterium*, *Lactobacillus*	16.99%	31.00%	17.53%	35.16%
*Corynebacterium*, *Brachybacterium*, *Joetgalibacillus/Salinicoccus*	13.48%	6.35%	11.91%	8.16%
*Lactobacillus*	18.90%	3.87%	19.86%	3.63%
*Staphylococcus* sp.	7.20%	4.75%	7.26%	3.62%
*Unknown*	11.65%	27.32%	12.11%	24.80%
*Yania*, *Clostridia*	9.21%	12.37%	10.45%	11.49%

* No significant differences in composition were observed between treatments using Tukey test (*P* > 0.05).

**Table 5:** Prevalence of resistance genes in litter obtained from 5 sequential flocks of broiler chickens raised on 3 commercial poultry farms. Two houses on each farm received no antibiotics; two houses received flavomycin or flavomycin + virginiamycin feed.

TREATMENT *		*ermA*			*ermB*			*ermC*			*tetM*			*tetO*			*vatA*			*vatB*			*vatD*		*vatE*			*vgaB*			*intI1*		
		Farm			Farm			Farm			Farm			Farm			Farm			Farm			Farm		Farm			Farm			Farm		
		1	2	3	1	2	3	1	2	3	1	2	3	1	2	3	1	2	3	1	2	3	1	2	1	2	3	1	2	3	1	2	3
**Flock 1**	Flavomycin	+	+	+	+	+	+	-	-	-	+	+	+	-	-	+	-	-	+	+	+	-	-	-	-	-	+	-	-	+	+	+	+
		+	+	+	+	+	+	-	-	-	-	+	+	-	-	+	-	-	+	-	+	-	-	-	-	-	+	-	-	+	+	+	+
	NONE	+	+	+	+	+	+	-	-	-	+	+	+	-	-	+	-	-	+	+	+	-	-	-	-	+	+	-	-	+	+	+	+
		+	+	+	+	+	+	-	-	-	+	+	+	-	-	+	-	-	+	-	+	-	-	-	-	+	+	-	-	+	+	+	+
**Flock 2**	Flavomycin + Virginiamycin	-	+	+	+	+	+	-	-	-	+	-	+	-	-	+	-	-	+	+	+	-	-	-	-	+	+	-	-	-	+	+	+
		+	+	+	+	+	+	-	-	-	+	+	+	-	-	+	-	-	+	-	+	-	-	-	-	-	+	-	-	+	+	+	+
	NONE	+	+	+	+	+	+	-	-	-	+	+	+	-	-	+	-	-	+	+	+	-	-	-	-	+	+	-	-	+	+	+	+
		+	+	+	+	+	+	-	-	-	+	+	+	-	-	+	-	-	+	-	+	-	-	-	-	-	+	-	-	+	+	+	+
**Flock 3**	Flavomycin + Virginiamycin	+	+	+	+	+	-	-	-	-	+	+	+	-	-	+	+	-	+	-	+	-	-	-	-	+	+	-	-	+	+	+	+
		+	+	+	+	+	+	-	-	-	+	+	+	-	-	+	-	-	+	-	-	-	-	-	-	+	+	-	-	+	+	+	+
	NONE	+	+	+	+	+	-	-	-	-	+	-	+	-	-	+	-	-	+	-	-	-	-	-	-	-	+	-	-	+	+	+	+
		+	+	+	+	+	+	-	-	-	+	-	+	-	-	+	-	-	+	-	-	-	-	-	-	-	+	-	-	+	+	+	+
**Flock 4**	Flavomycin + Virginiamycin	+	+	+	+	+	+	-	-	-	+	+	+	-	-	+	-	-	+	-	-	-	-	-	-	-	+	-	-	+	-	+	+
		+	+	+	+	+	+	-	-	-	+	-	+	-	-	+	-	-	+	-	-	-	-	-	-	-	+	-	-	+	+	+	+
	NONE	+	-	+	+	+	+	-	-	-	-	-	+	-	-	+	-	-	+	-	-	-	-	-	-	+	+	-	-	+	+	+	+
		-	-	+	+	+	+	-	-	-	+	+	+	-	-	+	-	-	+	-	+	-	-	-	-	-	+	-	-	+	+	+	+
**Flock 5**	Flavomycin	-	+	+	-	+	+	-	-	-	+	+	+	-	-	+	-	-	+	+	+	-	-	-	-	-	-	-	-	+	+	+	+
		-	+	+	-	+	+	-	-	-	+	+	+	-	-	+	-	-	+	-	+	-	-	-	-	+	+	-	-	+	+	+	+
	NONE	-	+	+	+	+	+	-	-	-	+	+	+	-	-	+	-	-	+	-	+	-	-	-	-	+	+	-	-	+	+	+	+
		+	-	+	+	+	+	-	-	-	+	+	+	-	-	+	-	-	+	-	+	-	-	-	-	+	-	-	-	+	+	+	+
**Total prevalence**	AGP	90%			90%			0%			90%			33%			33%			33%			0%		43%			30%			97%		
	NONE	83%			97%			0%			87%			33%			33%			30%			0%		50%			33%			100%		

* No significant differences were observed among treatments using Cochran-Armitage test (*P* > 0.05).

Class 1 integons, *ermA*, *ermB*, and *tetM* were detected in nearly every sample (83%–100% prevalence) while *tetO*, *vatA and vgaB* were primarily detected on farm 3. No *ermC* or *vatD* were detected in any sample.

### 3.3. Composition of Poultry Litter in Research Facilities Housing Birds Treated with Prebiotics or Probiotics

**Figure 2:**
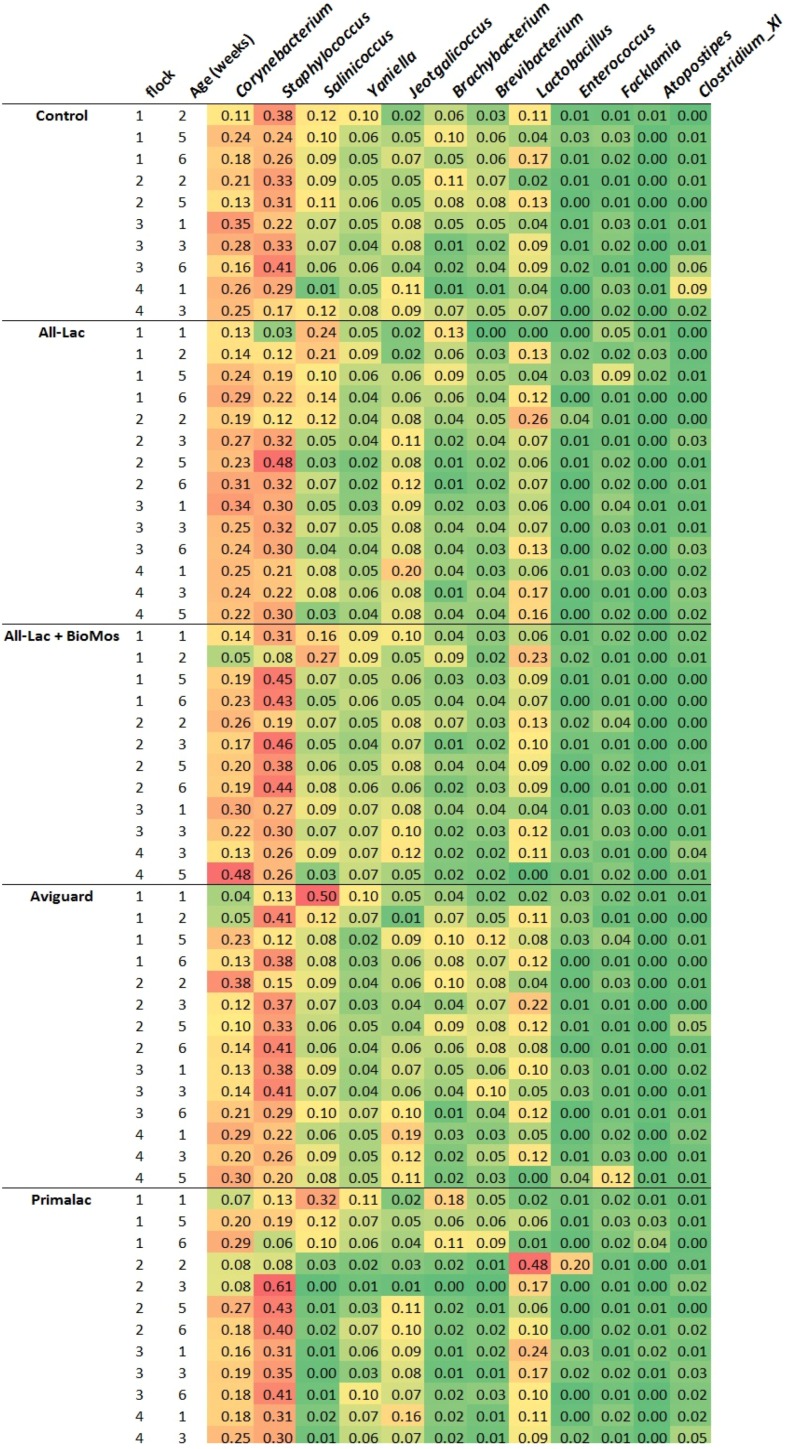
Abundance of genera contributing greater than 1% to total number of 16S rRNA sequences of 4 sequential flocks of broiler chickens administered probiotics or prebiotics and reared in research housing. The abundances are shown as the proportion of total sequences for each flock 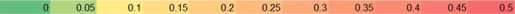.

Because no significant changes in litter community structure were seen associated with five sequential commercial flocks grown without antibiotics, we sought to determine if litter could be changed by bacteriotherapy of the birds themselves. The flocks were administered commercial products containing bacteria or mannan oligosaccharides which are marketed to improve intestinal microbial ecology, reduce intestinal disease, and improve feed conversion and reduce *Salmonella* prevalence. We hypothesized that they may also affect the microbial ecology of litter therefore litter community DNA was isolated and subjected to pyrosequencing. After processing of 16S sequences for quality and length, 64 libraries produced 367,688 sequences with an average length of 316 bp. Individual library sizes ranged from 434 to 42,046 sequences. Seven samples failed to generate a significant number of reads, thus were removed from the study analysis. A total of 1,103 operational taxonomic units (OTUs) at the species level were obtained. The majority of OTUs contributed less than 10 total sequences however the genera that were detected were primarily members of the phyla *Firmicutes* and *Actinobacteria* (Table 6 and Figure 2) with *Firmicutes* contributing an average of 58% and *Actinobacteria* contributing an average of 28%. There were many genera in which only one or two sequences were detected which is a common finding [63]. While each treatment group had 9–15 different genera contributing a minimum of 1% of sequences to the total, in regards to the overall composition of samples, the Primalac and All-Lac + BioMos groups were the most diverse with greater than 150 genera detected. The control group was the least diverse with only 85 different genera detected. 

The bacterial community of the first sample from the first cycle was statistically different (*P* < 0.05) from the last sample from the fourth cycle for all groups. The fact that the control house was statistically different indicates that time has a significant impact on the litter bacterial community. Table 7 shows the effect of time and treatments on litter composition. The first sample from all groups was dominated by the phylum Firmicutes however there was some variation in the most abundant genus. In the control group and All-Lac + BioMos treatment, *Staphylococcus* was most abundant while the other groups were dominated by *Salinococcus*. Firmicutes were also the most abundant phylum in the last sample in all treatment groups except the flocks receiving All-Lac + BioMos, which were dominated by the phylum Actinobacteria. Over time, only the Primalac group did not exhibit an increase in Actinobacteria. A decrease in the Firmicutes/Actinobacteria ratio from the first to last sample was observed in the control group compared to the average of the ratio observed in the litter from treated chickens (1.57 *versus* 1.87). This observation suggests that continued administration of bacteriotherapy may be necessary to maintain the abundance of the Firmicutes in the litter. *Corynebacterium*, *Brachybacterium* and *Brevibacterium* were the most abundant genera from the phylum Actinobacteria. This shift in bacterial communities over multiple production cycles is not unexpected given the physical differences between fresh and used litter. Past studies have shown that members of the phylum Actinobacteria may be involved in the decomposition of organic material such as the wood shavings used as bedding in this study [64]. 

**Table 6:** Abundance and prevalence of genera comprising litter bacterial community of broiler chickens raised in research housing and administered probiotics or prebiotics. Number of 16S rRNA sequences exhibiting 97% similarity to each genus is shown with the percentage of sequences in parentheses.

Genera	Control	All-Lac	All-Lac + BioMos	Aviguard	Primalac
Total sequences	19,806	39,357	91,219	48,226	76,363
Number of genera	85	135	154	119	211
Staphylococcus *	5,605 (28.3)	9,603 (24.4)	32,171 (35.3)	14,648 (30.4)	19,138 (25.1)
Corynebacterium **	4,222 (21.3)	9,483 (24.1)	16,872 (18.5)	7,839 (16.3)	13,259 (17.4)
Lactobacillus *	1,955 (9.9)	3,925 (10.0)	9,424 (10.3)	4,767 (9.9)	7,775 (10.2)
Salinicoccus *	1,851 (9.3)	3,412 (8.7)	8,407 (9.2)	5,802 (12.0)	5,274 (6.9)
Yaniella *	1,272 (6.4)	1,796 (4.6)	5,610 (6.2)	2,467 (5.1)	4,793 (6.3)
Brachybacterium **	1,077 (5.4)	1,796 (4.6)	3,534 (3.9)	2,698 (5.6)	3,066 (4.0)
Brevibacterium **	948 (4.8)	1,371 (3.5)	2,666 (2.9)	2,758 (5.7)	2,660 (3.5)
Facklamia *	337 (1.7)	1,291 (3.3)	1,640 (1.8)	796 (1.7)	1,498 (2.0)
Clostridium XI *	295 (1.5)	424 (1.1)	0 (0.0)	556 (1.2)	1,091 (1.4)
Enterococcus *	0 (0.0)	548 (1.4)	0 (0.0)	623 (1.3)	974 (1.3)
Atopostipes *	0 (0.0)	0 (0.0)	0 (0.0)	0 (0.0)	1,345 (1.8)
Streptococcus *	0 (0.0)	0 (0.0)	0 (0.0)	0 (0.0)	1,260 (1.7)

* Firmicutes; ** Actinobacteria.

**Table 7:** 16S rRNA composition of first and last litter sample from broiler chickens raised in research housing and administered probiotics or prebiotics.

Treatment	Sample	Number of sequences	Number of genera	Shannon Diversity Index	*Firmicutes* (%)	*Actinobacteria* (%)	*Proteobacteria* (%)	Most abundant genus
**Control**	First	3181	40	2.43 (±0.05)	78.2	21.8	0.00	*Staphylococcus* (37.6%)
	Last	3068	43	2.72 (±0.05)	61	38.9	0.00	*Corynebacterium* (24.7%)
**All-Lac**	First	2244	57	3.10 (±0.14)	68.6	25.4	4.50	*Salinicoccus* (24.4%)
	Last	2888	25	2.36 (±0.05)	68.7	30.8	0.00	*Staphylococcus* (30.4%)
**All-Lac + BioMos**	First	8949	72	2.59 (±0.03)	78.1	21.8	0.05	*Staphylococcus* (30.7%)
	Last	587	24	1.89 (±0.13)	47.4	52.6	0.00	*Corynebacterium* (48%)
**Aviguard**	First	4480	42	2.56 (±0.03)	88.4	11.5	0.04	*Salinicoccus* (49.9%)
	Last	4468	42	2.40 (±0.03)	64.1	35.1	0.72	*Corynebacterium* (30%)
**Primalac**	First	2938	37	2.75 (±0.05)	66.4	32.5	0.27	*Salinicoccus* (32%)
	Last	2660	34	2.58 (±0.04)	70.8	28	0.56	*Staphylococcus* (30%)

In order to examine the effect of the treatments on the litter composition, the sequences from the first sample from cycle one and the last sample from cycle 4 were compared to the corresponding samples in the control group. Treatment groups receiving All-Lac and Aviguard were found to be statistically different from the control group first sample (*p* < 0.001 and *p* < 0.0001, respectively). When examining the effect of treatment on the litter community from the last sample from cycle four, treatment groups receiving All-Lac + BioMos, Aviguard and Primalac were significantly different than the control group (*p* < 0.0055, *p* < 0.0001, *p* < 0.0151, respectively). 

**Figure 3:**
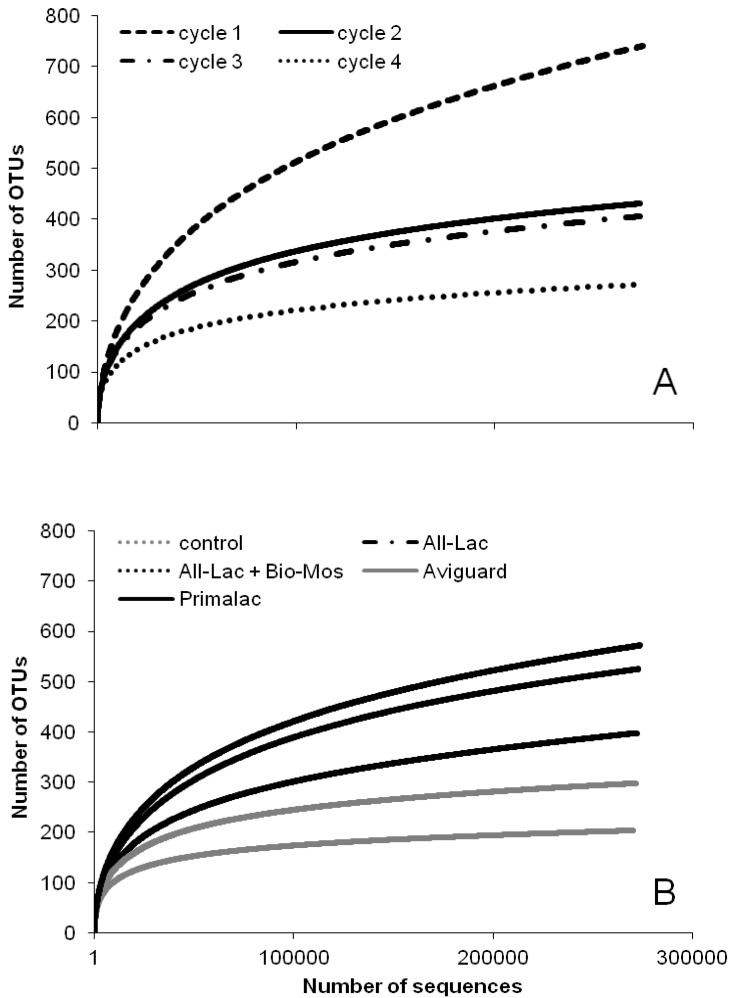
Rarefaction curve for bacterial 16S rRNA gene OTUs at 97% of similarity for litter samples collected from 4 sequential flocks of broiler chickens administered probiotics or prebiotics and reared in research housing. Graph (**A**) analyzed by flock; graph (**B**) by treatment.

**Figure 4:**
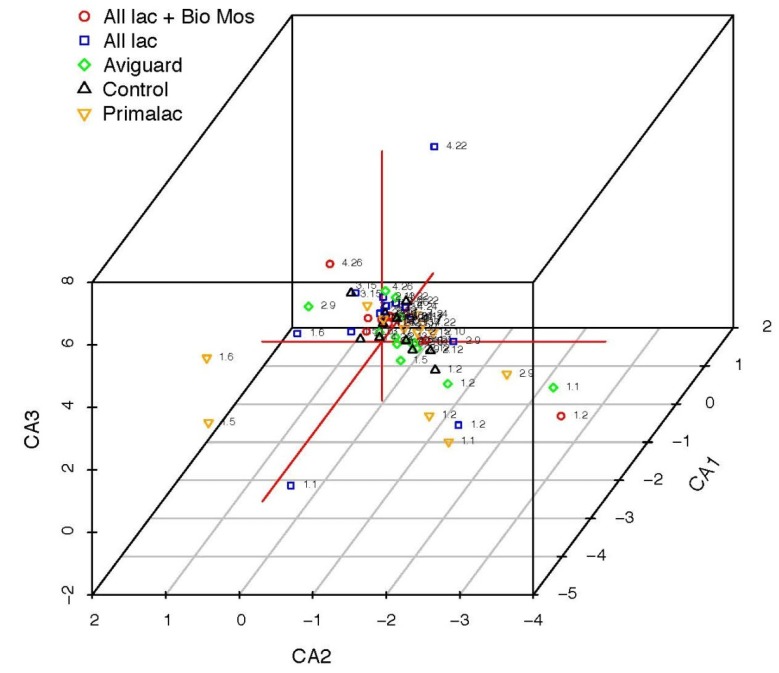
Correspondence analysis of microbial community patterns generated by 16S rRNA analysis of litter samples collected from four sequential flocks of broiler chickens administered probiotics or prebiotics and reared in research housing. Each point represents a single sample; the five treatments are represented by color and symbol type as shown in the legend. Labels for each point indicate litter cycle (1–4), followed by week of sample collection (1–26).

The Shannon diversity index was also used to compare composition of the litter community. The Shannon diversity index takes into consideration the different genera in a sample as well as the frequency of each species to quantify the entropy or uncertainty in a sample [65]. In other words, it quantifies the uncertainty of predicting a particular genus from a given sample, the greater the abundance, the more difficult it is to predict the next genera sampled. If there was only one genus present the Shannon diversity index would be zero because there is no difficulty in predicting the correct genera. Using this ecological parameter, the control group increased in diversity from the first sample to the last sample while all of the treatment groups actually decreased in diversity (Table 7). A rarefaction curve comparing the occurrence of different sequences, also called operational taxonomic units (OTUs), demonstrated that diversity decreased over time (Figure 3(A)) probably due to changes in the physical/chemical characteristics of litter. Treatment comparisons using OTU rarefaction indicated richness was higher in litter from treated chickens and lower in the control group (Figure 3(B)). This indicates that the all of the treatments have the ability to impact composition of the litter bacterial community. Canonical Correspondence Analysis (Figure 4) shows that the litter community is strongly affected by its age. Most outliers observed were samples collected in the beginning of the experiment, which possessed a low abundance of *Actinobacteria* and low diversity, and litter from chickens receiving Primalac which had the highest diversity. 

### 3.4. Effect of Time and Probiotic or Prebiotic Treatments on Pathogen Abundance in Litter

The litter DNA sequences were assessed for the presence of pathogenic species of bacteria and revealed that *Staphylococcus aureus* and *Clostridium perfringens* abundance was not affected by treatments or the cycle (data not shown). Abundance of *Clostridium sordellii* increased in litter over time but treatments did not affect abundance. Because *Salmonella* is known to contaminate poultry litter and it cannot be reliably detected using 16S rRNA, we screened multiple litter samples collected after the birds were 3 weeks of age from every flock by *Salmonella*-specific PCR in order to determine whether treatments decreased the prevalence of this pathogen (Table 8). *Salmonella* was detected within litter of every treatment group except flocks treated with Primalac. But, only the last flock of the All-Lac + BioMos group was *Salmonella*-positive while the other 3 groups had 50%–75% of flocks positive. These results indicate that bacteriotherapy can not only change the composition of poultry litter but can reduce the prevalence of pathogens. 

**Table 8:** Prevalence of *Salmonella* in litter of 4 sequential flocks of broiler chickens administered probiotics or prebiotics and reared in research housing.

Flock	Control	All-Lac	All-Lac + BioMos	Aviguard	Primalac
**1**	+	-	-	+	-
**2**	+	+	-	-	-
**3**	-	+	-	+	-
**4**	-	+	+	+	-

### 3.5. Effects of Probiotics and Prebiotics on the Prevalence of Antibiotic Resistance within the Bacterial Community of Poultry Litter

**Figure 5:**
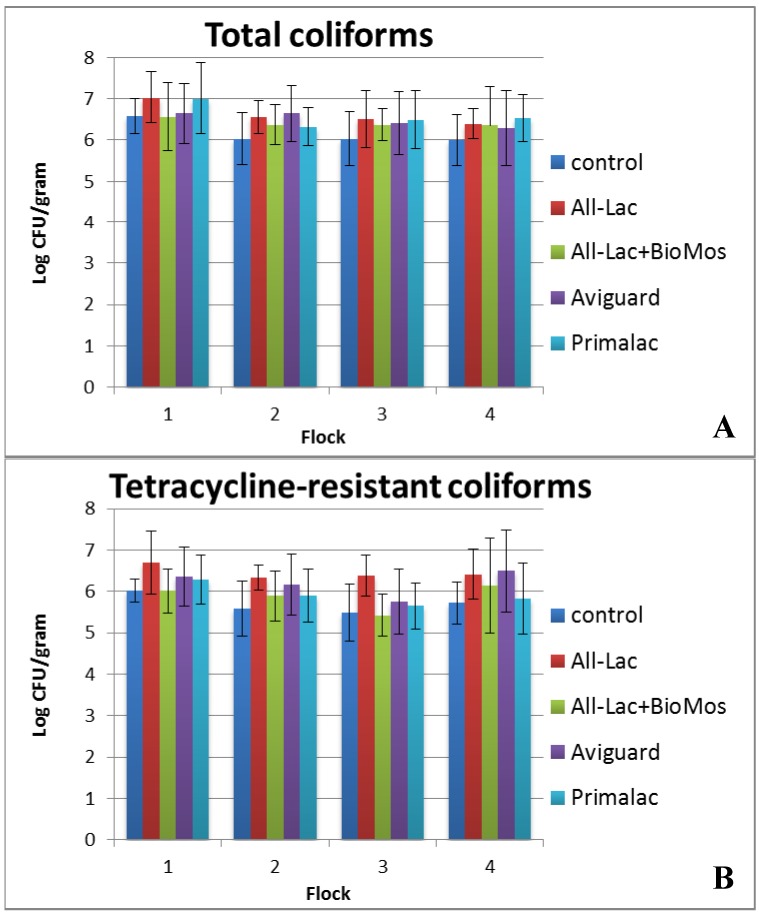
Log colony forming units (CFU) of coliforms (panel **A**) and tetracycline-resistant coliforms (panel **B**) cultured from litter collected from four sequential flocks of broiler chickens administered probiotics or prebiotics and reared in research housing.

Two approaches were used to assay for changes in the litter resistome. Because tetracycline resistance has been shown to be high among poultry *E. coli* [66] and high levels of *tetM* were found in commercial poultry litter, we were interested in whether bacteriotherapy of birds would decrease the abundance of tetracycline-resistant coliforms. We quantified the abundance of resistant and susceptible coliforms for each litter sample by plating on selective agar containing antibiotic. Figure 5 shows the abundance of resistant coliforms nearly equals the abundance of susceptible isolates for each treatment and over every flock cycle. There were no statistically significant differences between treatments in total amounts of coliforms, amounts of tetracycline resistant coliforms, or the ratio of tetracycline resistant coliforms to total coliforms in the litter or the fecal samples (data not shown) for any of the treatments. Common belief is that competition resulting from bacteriotherapy would reduce the abundance of antibiotic-resistant organisms or genes because of the potential fitness cost of resistance [67]. However, it may be that the tetracycline resistance genes have no cost for these bacteria, or there has been compensatory adaptation to ameliorate the cost [67,68]. Therefore we assayed for the abundance of another common resistance element, the class 1 integron, because it has been found in a diversity of bacteria including the most abundant members of the litter community [16,69]. Integron abundance was determined by quantitative real-time PCR on litter community DNA and the signal normalized to eubacterial genome copies. The mean *intI1*/eubacterial genome ratio ranged from 0.028–0.047 suggesting that 3%–5% of the total litter community of each treatment group contained an integron (Table 9). There were no significant differences detected in abundance over time, flock or treatment suggesting that integron carriage is stable in poultry litter. 

No significant differences were observed among treatments (*P* > 0.05).

**Table 9:** Prevalence and abundance of class 1 integron (*intI1*) genes in litter obtained from four sequential flocks of broiler chickens administered probiotics or prebiotics and reared in research housing. The number (#) of eubacterial genomes and *intI1* copies per 25 ng litter DNA was calculated after normalizing the quantitative PCR signal using a control strain and adjusting the signal to reflect genome numbers for the most abundant genera in broiler litter.

Treatment	# *intI1* copies * ± SD	# eubacterial genomes * ± SD	*intI1*: eubacterial genome ratio *	Mean Ratio ± SD
**Control** Flock 1	6.30 × 10^7^ ± 9.69 × 10^6^	1.15 × 10^9^ ± 1.23 × 10^7^	0.0547	
Flock 2	2.69 × 10^7^ ± 2.90 × 10^6^	1.26 × 10^9^ ± 3.03 × 10^8^	0.0214	
Flock 3	3.11 × 10^6^ ± 1.77 × 10^5^	5.25 × 10^8^ ± 1.05 × 10^7^	0.0059	
Flock 4	2.97 × 10^7^ ± 8.46 × 10^6^	1.04 × 10^9^ ± 5.30 × 10^7^	0.0286	0.0277 ± 0.0204
**All-Lac** Flock 1	3.30 × 10^7^ ± 5.87 × 10^5^	1.10 × 10^9^ ± 2.54 × 10^8^	0.0300	
Flock 2	1.59 × 10^7^ ± 9.31 × 10^6^	5.23 × 10^8^ ± 2.74 × 10^7^	0.0304	
Flock 3	3.15 × 10^7^ ± 6.79 × 10^6^	1.29 × 10^9^ ± 2.25 × 10^8^	0.0244	
Flock 4	4.41 × 10^7^ ± 7.29 × 10^6^	2.07 × 10^9^ ± 1.06 × 10^9^	0.0213	0.0265 ± 0.0044
**All-Lac + BioMos** Flock 1	2.31 × 10^7^ ± 4.12 × 10^6^	7.26 × 10^8^ ± 3.76 × 10^7^	0.0318	
Flock 2	3.38 × 10^7^ ± 1.44 × 10^6^	1.28 × 10^9^ ± 1.29 × 10^8^	0.0263	
Flock 3	2.81 × 10^7^ ± 2.09 × 10^6^	1.10 × 10^9^ ± 3.63 × 10^7^	0.0254	
Flock 4	2.37 × 10^7^ ± 4.03 × 10^6^	1.05 × 10^9^ ± 4.58 × 10^7^	0.0224	0.0265 ± 0.0039
**Aviguard** Flock 1	3.25 × 10^7^ ± 1.06 × 10^6^	1.14 × 10^9^ ± 2. 06 × 10^8^	0.0286	
Flock 2	3.56 × 10^6^ ± 2.02 × 10^5^	7.80 × 10^7^ ± 4.63 × 10^6^	0.0456	
Flock 3	5.98 × 10^7^ ± 3.42 × 10^6^	8.17 × 10^8^ ± 2.67 × 10^7^	0.0732	
Flock 4	4.18 × 10^7^ ± 2.91 × 10^6^	1.06 × 10^9^ ± 2.16 × 10^8^	0.0393	0.0467 ± 0.0190
**Primalac** Flock 1	5.69 × 10^7 ^± 2.06 × 10^7^	7.06 × 10^8^ ± 6.80 × 10^7^	0.0806	
Flock 2	6.94 × 10^7^ ± 2.61 × 10^7^	1.33 × 10^9^ ± 4.24 × 10^5^	0.0520	
Flock 3	2.76 × 10^7^ ± 3.54 × 10^5^	8.64 × 10^8^ ± 6.04 × 10^7^	0.0320	
Flock 4	1.12 × 10^7^ ± 7.07 × 10^3^	4.27 × 10^8^ ± 5.83 × 10^7^	0.0263	0.0477 ± 0.0245

* No significant differences were observed among treatments using Tukey test (*P* > 0.05).

## 4. Conclusions

The deep litter system, where poultry litter is reused during consecutive cycles as a practice to reduce environmental waste, is common in many countries. However little is known about the impact of time or flock treatments on the microbial composition of poultry litter. Poultry litter under correct management is partially responsible for good animal production indices [70], because it acts as a source of beneficial organisms to colonize the intestinal tract of neonatal poultry which do not have exposure to the microbiota of adult birds [71]. Litter management is essential for disease control within the industry [72]. However, poultry litter has been shown to contain a diversity of pathogens, heavy metals, and have high levels of antibiotic resistance [14,16,19,73,74,75,76,77,78,79]. Studies investigating the stability of resistant organisms and resistance genes within litter have shown that some persist for long periods of time in stored litter suggesting that traditional storage methods may have little benefit [74,75,78]. In fact several studies have revealed increased prevalence and abundance of resistant organisms and resistance genes in soil that has been amended with litter or animal manure [11,15,18,80,81,82]. In this study we confirmed the high prevalence of resistance genes despite the absence of antibiotic selective pressure in multiple sequential flocks raised in the same poultry house. Using large scale libraries of litter 16S rRNA, from four cycles of antibiotic-free production with commercial alternatives to growth promoting antibiotics, we investigated whether these production changes would affect litter composition, abundance of antibiotic resistance and frequency of pathogens. Probiotics and prebiotics demonstrated the ability to alter the litter microbial community and some treatments reduced the prevalence of *Salmonella* in the litter. While the treatments changed the composition of the litter community, they did not reduce the prevalence or abundance of tetracycline-resistant *E. coli* or the class 1 integron resistance element, which is commonly detected in human and animal pathogens such as *Salmonella*, *E. coli*, *Shigella*, among others [69]. Application of litter and manures to land on which organic fruits and vegetables are produced could affect the prevalence of resistant bacteria on organic foods [83,84,85]. It is imperative that we identify methods to mitigate the abundance of resistance genes in litter and manures, however the full benefits of prebiotics and probiotics may require many, many cycles of production in order to affect the farm environment. Realizing environmental improvements from the usage of alternatives to antibiotics is in its infancy, as well as the study of the microbial ecology of broiler chicken production and its impact on the environment. Future research is needed to fully utilize these products in an effort to improve the environmental microbiome and resistome of food animal production.

## Supplementary Files

Supplementary File 1 Support Information 1 (PDF, 2526 KB)
