# Supplementary material for: Can Probiotics Improve the Environmental Microbiome and Resistome of Commercial Poultry Production?
Source: Int J Environ Res Public Health. 2013 Sep 25;10(10):4534–59. doi: 10.3390/ijerph10104534 (PMC3823317; doi:10.3390/ijerph10104534)
Supplement: Supplementary File 1 — Support Information 1 (PDF, 2526 KB) [file ijerph-10-04534-s001.pdf]

Supplementary Information

Can Probiotics Improve the Environmental Microbiome and Resistome of Commercial Poultry Production?

Abundance of T-RFLP peaks contributing greater than 2% of total 16S rRNA signal of litter from 5 sequential flocks of broiler chickens reared on commercial poultry farms. Two houses (C and D) on each farm contained birds that were fed antibiotic growth promoters (AGP); houses A and B were fed no antibiotics. Red → green gradient in the columns shows the proportion of total sample signal represented by each fragment. 0 | 0.05 | 0.1 | 0.15 | 0.2 | 0.25 | 0.3 | 0.35 | 0.4 | 0.45 | 0.5

	FARM 1										FARM 1 - AGP								
	HOUSE A					HOUSE B					HOUSE C					HOUSE D			
T-RFLP Size (bp)	FLOCK 1	FLOCK 2	FLOCK 3	FLOCK 4	FLOCK 5	FLOCK 1	FLOCK 2	FLOCK 3	FLOCK 4	FLOCK 5	FLOCK 1	FLOCK 2	FLOCK 3	FLOCK 4	FLOCK 5	FLOCK 1	FLOCK 2	FLOCK 3	FLOCK 5
63	0.05	0.06	0.07	0.06	0.07	0.06	0.04	0.06	0.00	0.32	0.06	0.04	0.07	0.07	0.10	0.04	0.06	0.08	0.05
64	0.00	0.00	0.00	0.00	0.00	0.00	0.00	0.00	0.00	0.00	0.00	0.00	0.00	0.00	0.00	0.00	0.00	0.00	0.00
65	0.00	0.00	0.00	0.00	0.00	0.02	0.00	0.00	0.00	0.00	0.00	0.00	0.00	0.00	0.00	0.01	0.00	0.00	0.00
68	0.00	0.00	0.00	0.00	0.00	0.00	0.00	0.00	0.00	0.00	0.00	0.00	0.00	0.00	0.00	0.00	0.00	0.00	0.00
69	0.00	0.00	0.00	0.00	0.00	0.00	0.00	0.00	0.00	0.00	0.00	0.00	0.00	0.00	0.06	0.00	0.00	0.00	0.00
71	0.00	0.01	0.00	0.00	0.00	0.00	0.00	0.00	0.00	0.00	0.02	0.04	0.02	0.00	0.00	0.04	0.00	0.00	0.00
77	0.00	0.02	0.03	0.00	0.00	0.02	0.00	0.00	0.00	0.00	0.02	0.03	0.03	0.00	0.00	0.03	0.00	0.00	0.00
78	0.07	0.06	0.07	0.10	0.07	0.05	0.08	0.14	0.17	0.00	0.04	0.05	0.07	0.12	0.11	0.04	0.09	0.08	0.10
81	0.00	0.00	0.00	0.00	0.00	0.00	0.00	0.00	0.00	0.00	0.00	0.01	0.00	0.00	0.00	0.01	0.00	0.00	0.00
178	0.02	0.05	0.07	0.08	0.07	0.04	0.07	0.05	0.04	0.00	0.06	0.04	0.07	0.05	0.10	0.04	0.09	0.09	0.06
181	0.02	0.05	0.06	0.07	0.09	0.04	0.00	0.06	0.05	0.00	0.05	0.00	0.07	0.06	0.00	0.04	0.02	0.06	0.04
182	0.00	0.00	0.00	0.00	0.00	0.00	0.00	0.00	0.00	0.00	0.00	0.05	0.00	0.00	0.09	0.00	0.00	0.00	0.00
199	0.00	0.00	0.00	0.00	0.00	0.00	0.00	0.00	0.00	0.00	0.00	0.00	0.00	0.00	0.00	0.00	0.00	0.00	0.00
201	0.00	0.02	0.02	0.02	0.02	0.02	0.00	0.00	0.00	0.00	0.00	0.04	0.02	0.00	0.00	0.03	0.00	0.00	0.00
205	0.00	0.00	0.00	0.00	0.00	0.00	0.00	0.00	0.00	0.00	0.00	0.00	0.00	0.00	0.00	0.01	0.00	0.00	0.00
206	0.00	0.00	0.00	0.00	0.00	0.00	0.00	0.00	0.00	0.00	0.00	0.01	0.00	0.00	0.00	0.00	0.00	0.00	0.00
208	0.00	0.00	0.00	0.00	0.00	0.00	0.00	0.00	0.00	0.00	0.00	0.01	0.00	0.00	0.00	0.00	0.00	0.00	0.00
210	0.00	0.01	0.01	0.00	0.00	0.01	0.00	0.00	0.00	0.00	0.01	0.02	0.00	0.00	0.00	0.02	0.00	0.00	0.00
212	0.00	0.01	0.02	0.00	0.00	0.01	0.00	0.00	0.00	0.00	0.01	0.03	0.01	0.00	0.00	0.03	0.00	0.00	0.00
215	0.00	0.00	0.00	0.00	0.00	0.00	0.00	0.00	0.00	0.00	0.00	0.00	0.00	0.00	0.00	0.01	0.00	0.00	0.00
217	0.00	0.00	0.00	0.00	0.00	0.00	0.00	0.00	0.00	0.00	0.00	0.00	0.00	0.00	0.00	0.01	0.00	0.00	0.00
219	0.00	0.00	0.00	0.00	0.00	0.00	0.00	0.00	0.00	0.00	0.00	0.01	0.00	0.00	0.00	0.00	0.00	0.00	0.00
221	0.00	0.00	0.00	0.00	0.00	0.00	0.00	0.00	0.00	0.11	0.00	0.01	0.00	0.00	0.00	0.00	0.00	0.00	0.00
222	0.00	0.00	0.00	0.00	0.00	0.00	0.00	0.00	0.00	0.00	0.00	0.00	0.00	0.00	0.00	0.01	0.00	0.00	0.00
224	0.00	0.00	0.01	0.00	0.01	0.05	0.00	0.00	0.00	0.00	0.00	0.00	0.00	0.00	0.00	0.00	0.00	0.00	0.00
225	0.00	0.00	0.00	0.00	0.00	0.00	0.00	0.00	0.00	0.00	0.00	0.01	0.00	0.00	0.03	0.02	0.00	0.00	0.00
226	0.00	0.00	0.00	0.00	0.00	0.00	0.00	0.00	0.00	0.00	0.00	0.00	0.00	0.00	0.00	0.00	0.00	0.00	0.00
227	0.00	0.00	0.00	0.00	0.00	0.00	0.00	0.00	0.00	0.00	0.00	0.00	0.00	0.00	0.00	0.00	0.00	0.00	0.00
228	0.00	0.00	0.00	0.00	0.00	0.00	0.00	0.00	0.00	0.00	0.00	0.00	0.00	0.00	0.00	0.00	0.00	0.00	0.00
229	0.00	0.00	0.00	0.00	0.00	0.00	0.00	0.00	0.00	0.00	0.00	0.00	0.00	0.00	0.00	0.00	0.00	0.00	0.00
230	0.06	0.00	0.00	0.00	0.00	0.00	0.00	0.09	0.20	0.00	0.00	0.00	0.00	0.16	0.00	0.00	0.00	0.00	0.12
231	0.00	0.00	0.00	0.00	0.00	0.00	0.00	0.00	0.00	0.00	0.00	0.00	0.00	0.00	0.00	0.00	0.00	0.00	0.00
232	0.00	0.00	0.00	0.00	0.00	0.00	0.00	0.00	0.00	0.28	0.00	0.00	0.00	0.00	0.00	0.00	0.00	0.00	0.00
233	0.08	0.06	0.07	0.09	0.10	0.06	0.08	0.15	0.22	0.00	0.06	0.00	0.08	0.16	0.00	0.00	0.10	0.10	0.12
234	0.00	0.00	0.00	0.08	0.06	0.00	0.03	0.00	0.00	0.13	0.00	0.05	0.07	0.00	0.10	0.00	0.00	0.00	0.00
235	0.00	0.00	0.00	0.00	0.00	0.00	0.00	0.00	0.00	0.00	0.00	0.00	0.00	0.00	0.00	0.04	0.00	0.00	0.00
236	0.00	0.00	0.02	0.00	0.03	0.00	0.00	0.00	0.00	0.00	0.02	0.03	0.00	0.00	0.00	0.00	0.00	0.02	0.00
237	0.00	0.02	0.00	0.03	0.00	0.03	0.00	0.00	0.00	0.00	0.00	0.00	0.03	0.00	0.05	0.03	0.00	0.00	0.00
238	0.01	0.00	0.00	0.00	0.00	0.00	0.00	0.00	0.00	0.00	0.00	0.00	0.00	0.00	0.00	0.00	0.00	0.00	0.00
241	0.00	0.00	0.00	0.00	0.00	0.00	0.00	0.13	0.07	0.00	0.00	0.00	0.00	0.05	0.00	0.00	0.00	0.00	0.00
242	0.08	0.00	0.05	0.08	0.09	0.06	0.10	0.00	0.00	0.16	0.05	0.04	0.07	0.00	0.05	0.04	0.00	0.10	0.12
243	0.00	0.00	0.00	0.00	0.00	0.00	0.00	0.00	0.00	0.00	0.00	0.00	0.00	0.00	0.00	0.00	0.00	0.00	0.00
244	0.00	0.04	0.06	0.02	0.00	0.00	0.00	0.03	0.00	0.00	0.03	0.03	0.00	0.00	0.00	0.04	0.00	0.03	0.00
245	0.00	0.03	0.00	0.00	0.00	0.00	0.02	0.00	0.00	0.00	0.00	0.00	0.00	0.00	0.00	0.00	0.00	0.00	0.00
246	0.02	0.00	0.00	0.02	0.02	0.04	0.05	0.04	0.00	0.00	0.03	0.03	0.04	0.00	0.00	0.00	0.04	0.04	0.00
247	0.09	0.00	0.00	0.00	0.00	0.00	0.00	0.00	0.00	0.00	0.00	0.00	0.00	0.00	0.00	0.00	0.00	0.00	0.05
248	0.00	0.07	0.05	0.03	0.03	0.06	0.03	0.00	0.00	0.00	0.05	0.05	0.03	0.03	0.04	0.04	0.07	0.03	0.00
249	0.00	0.01	0.02	0.00	0.00	0.00	0.00	0.00	0.00	0.00	0.00	0.01	0.00	0.00	0.00	0.00	0.02	0.00	0.00
250	0.00	0.00	0.00	0.00	0.00	0.00	0.00	0.00	0.00	0.00	0.02	0.00	0.00	0.00	0.00	0.02	0.00	0.00	0.00
251	0.00	0.01	0.01	0.00	0.00	0.00	0.00	0.00	0.00	0.00	0.00	0.01	0.00	0.00	0.00	0.02	0.02	0.00	0.00
255	0.00	0.00	0.00	0.00	0.00	0.00	0.00	0.00	0.00	0.00	0.00	0.00	0.00	0.00	0.00	0.01	0.00	0.00	0.00
261	0.00	0.00	0.00	0.00	0.00	0.00	0.00	0.00	0.00	0.00	0.00	0.01	0.00	0.00	0.00	0.01	0.00	0.00	0.00
264	0.02	0.00	0.00	0.00	0.00	0.00	0.05	0.00	0.00	0.00	0.00	0.00	0.00	0.00	0.00	0.00	0.04	0.00	0.00
265	0.00	0.06	0.03	0.00	0.00	0.03	0.00	0.00	0.00	0.00	0.03	0.04	0.02	0.00	0.00	0.03	0.10	0.00	0.00
266	0.09	0.06	0.07	0.05	0.06	0.06	0.10	0.06	0.05	0.00	0.05	0.05	0.07	0.07	0.05	0.04	0.00	0.10	0.09
271	0.00	0.00	0.00	0.00	0.00	0.01	0.00	0.00	0.00	0.00	0.03	0.00	0.00	0.00	0.00	0.01	0.00	0.00	0.00
273	0.08	0.02	0.00	0.00	0.00	0.02	0.00	0.00	0.00	0.00	0.02	0.00	0.00	0.00	0.00	0.01	0.00	0.00	0.00
275	0.00	0.01	0.00	0.00	0.00	0.02	0.00	0.00	0.00	0.00	0.01	0.01	0.00	0.00	0.00	0.02	0.00	0.00	0.00
278	0.00	0.00	0.00	0.00	0.00	0.00	0.00	0.00	0.00	0.00	0.00	0.00	0.02	0.00	0.00	0.00	0.00	0.00	0.00
279	0.02	0.03	0.03	0.00	0.02	0.04	0.00	0.00	0.00	0.00	0.02	0.02	0.00	0.00	0.00	0.02	0.00	0.00	0.03
283	0.00	0.01	0.00	0.02	0.00	0.00	0.00	0.00	0.00	0.00	0.01	0.02	0.00	0.00	0.03	0.02	0.00	0.00	0.00
284	0.00	0.00	0.00	0.00	0.00	0.00	0.00	0.00	0.00	0.00	0.00	0.00	0.00	0.00	0.00	0.00	0.00	0.00	0.00
285	0.00	0.00	0.00	0.02	0.00	0.02	0.00	0.00	0.00	0.00	0.00	0.01	0.00	0.00	0.03	0.02	0.00	0.00	0.00
288	0.02	0.03	0.00	0.00	0.00	0.04	0.03	0.00	0.00	0.00	0.03	0.01	0.02	0.00	0.00	0.00	0.00	0.00	0.00
294	0.00	0.00	0.00	0.00	0.00	0.00	0.00	0.00	0.00	0.00	0.00	0.00	0.00	0.00	0.00	0.01	0.00	0.00	0.00
298	0.00	0.00	0.00	0.00	0.00	0.00	0.00	0.00	0.00	0.00	0.00	0.01	0.00	0.00	0.00	0.01	0.00	0.00	0.00
301	0.00	0.00	0.00	0.00	0.00	0.01	0.00	0.00	0.00	0.00	0.01	0.02	0.00	0.00	0.01	0.02	0.00	0.00	0.00
305	0.00	0.00	0.00	0.00	0.00	0.00	0.00	0.00	0.00	0.00	0.00	0.00	0.00	0.00	0.00	0.00	0.00	0.00	0.00
306	0.05	0.00	0.00	0.08	0.08	0.00	0.10	0.08	0.07	0.00	0.06	0.00	0.08	0.08	0.08	0.00	0.09	0.08	0.05
307	0.00	0.00	0.00	0.04	0.00	0.00	0.00	0.00	0.00	0.00	0.05	0.00	0.00	0.00	0.00	0.00	0.00	0.00	0.06
308	0.04	0.00	0.00	0.00	0.06	0.00	0.05	0.00	0.04	0.00	0.00	0.00	0.00	0.00	0.00	0.00	0.06	0.05	0.00
309	0.06	0.00	0.00	0.10	0.00	0.00	0.00	0.00	0.00	0.00	0.00	0.00	0.00	0.00	0.03	0.00	0.00	0.09	0.08
310	0.00	0.07	0.07	0.00	0.09	0.06	0.10	0.08	0.09	0.00	0.06	0.05	0.08	0.12	0.00	0.03	0.10	0.00	0.00
313	0.00	0.00	0.00	0.00	0.00	0.00	0.00	0.00	0.00	0.00	0.00	0.00	0.00	0.00	0.00	0.02	0.00	0.00	0.00
314	0.00	0.00	0.00	0.00	0.00	0.00	0.00	0.00	0.00	0.00	0.00	0.01	0.00	0.00	0.00	0.00	0.00	0.00	0.00
316	0.09	0.07	0.07	0.00	0.02	0.06	0.03	0.00	0.00	0.00	0.00	0.00	0.01	0.00	0.02	0.00	0.04	0.04	0.04
317	0.00	0.00	0.00	0.00	0.00	0.00	0.00	0.00	0.00	0.00	0.06	0.05	0.00	0.00	0.00	0.04	0.00	0.00	0.00
319	0.00	0.00	0.01	0.00	0.00	0.00	0.00	0.00	0.00	0.00	0.00	0.00	0.00	0.00	0.00	0.00	0.00	0.00	0.00
321	0.00	0.02	0.00	0.00	0.00	0.00	0.01	0.00	0.00	0.00	0.01	0.00	0.00	0.00	0.00	0.02	0.01	0.00	0.00
322	0.00	0.00	0.01	0.00	0.00	0.00	0.00	0.00	0.00	0.00	0.00	0.01	0.00	0.00	0.00	0.00	0.00	0.00	0.00
323	0.01	0.00	0.00	0.00	0.00	0.02	0.00	0.00	0.00	0.00	0.00	0.00	0.00	0.00	0.00	0.00	0.00	0.00	0.00
324	0.00	0.00	0.00	0.00	0.00	0.00	0.00	0.00	0.00	0.00	0.00	0.00	0.00	0.00	0.00	0.00	0.00	0.00	0.00
325	0.00	0.00	0.00	0.00	0.00	0.00	0.00	0.00	0.00	0.00	0.00	0.00	0.00	0.00	0.00	0.00	0.00	0.00	0.00
328	0.00	0.00	0.00	0.00	0.00	0.00	0.00	0.00	0.00	0.00	0.02	0.00	0.00	0.00	0.00	0.00	0.00	0.00	0.00
329	0.00	0.03	0.03	0.03	0.02	0.03	0.02	0.03	0.00	0.00	0.00	0.05	0.03	0.03	0.02	0.04	0.02	0.02	0.00
330	0.00	0.00	0.00	0.00	0.00	0.00	0.00	0.00	0.00	0.00	0.00	0.00	0.00	0.00	0.00	0.00	0.00	0.00	0.00
331	0.02	0.03	0.02	0.00	0.00	0.04	0.02	0.00	0.00	0.00	0.01	0.02	0.00	0.00	0.00	0.02	0.02	0.00	0.00
345	0.00	0.00	0.00	0.00	0.00	0.00	0.00	0.00	0.00	0.00	0.00	0.00	0.00	0.00	0.00	0.00	0.00	0.00	0.00
373	0.00	0.00	0.00	0.00	0.00	0.00	0.00	0.00	0.00	0.00	0.00	0.00	0.00	0.00	0.00	0.00	0.00	0.00	0.00

T-RFLP Size (bp)	FARM 2										FARM 2 - AGP									
	HOUSE A					HOUSE B					HOUSE C					HOUSE D				
	FLOCK 1	FLOCK 2	FLOCK 3	FLOCK 4	FLOCK 5	FLOCK 1	FLOCK 2	FLOCK 3	FLOCK 4	FLOCK 5	FLOCK 1	FLOCK 2	FLOCK 3	FLOCK 4	FLOCK 5	FLOCK 1	FLOCK 2	FLOCK 3	FLOCK 4	FLOCK 5
63	0.25	0.13	0.18	0.20	0.17	0.27	0.20	0.16	0.23	0.18	0.24	0.17	0.21	0.19	0.16	0.17	0.21	0.17	0.23	0.13
64	0.00	0.00	0.00	0.00	0.00	0.00	0.00	0.00	0.00	0.05	0.00	0.00	0.00	0.00	0.00	0.00	0.00	0.00	0.00	0.00
65	0.00	0.00	0.00	0.00	0.00	0.00	0.00	0.00	0.00	0.00	0.00	0.00	0.00	0.00	0.00	0.00	0.00	0.00	0.00	0.00
68	0.00	0.00	0.00	0.00	0.00	0.00	0.00	0.00	0.00	0.00	0.00	0.00	0.00	0.00	0.00	0.00	0.00	0.00	0.00	0.00
69	0.01	0.00	0.01	0.00	0.01	0.01	0.00	0.01	0.01	0.00	0.00	0.00	0.00	0.01	0.00	0.01	0.00	0.01	0.01	0.01
71	0.00	0.00	0.00	0.00	0.00	0.00	0.00	0.00	0.00	0.00	0.00	0.00	0.00	0.00	0.00	0.00	0.00	0.00	0.00	0.00
77	0.00	0.00	0.00	0.00	0.00	0.00	0.00	0.00	0.00	0.00	0.00	0.00	0.00	0.00	0.01	0.00	0.00	0.00	0.00	0.00
78	0.10	0.15	0.10	0.16	0.07	0.10	0.09	0.11	0.07	0.05	0.18	0.24	0.17	0.13	0.15	0.16	0.15	0.12	0.08	0.15
81	0.00	0.00	0.00	0.00	0.00	0.00	0.00	0.00	0.00	0.00	0.00	0.00	0.00	0.00	0.00	0.00	0.00	0.00	0.00	0.00
178	0.03	0.05	0.07	0.13	0.10	0.03	0.07	0.07	0.11	0.15	0.03	0.08	0.11	0.16	0.11	0.01	0.07	0.03	0.07	0.10
181	0.06	0.08	0.04	0.02	0.07	0.06	0.00	0.06	0.00	0.00	0.06	0.08	0.03	0.02	0.03	0.00	0.08	0.00	0.06	0.02
182	0.00	0.00	0.00	0.00	0.00	0.00	0.07	0.00	0.02	0.04	0.00	0.00	0.00	0.00	0.00	0.07	0.00	0.08	0.00	0.00
199	0.00	0.00	0.00	0.00	0.00	0.00	0.00	0.00	0.00	0.00	0.00	0.00	0.00	0.00	0.00	0.00	0.00	0.00	0.01	0.00
201	0.00	0.00	0.00	0.00	0.00	0.00	0.00	0.00	0.00	0.00	0.00	0.00	0.00	0.00	0.00	0.00	0.00	0.00	0.00	0.00
205	0.00	0.00	0.00	0.00	0.00	0.00	0.00	0.00	0.00	0.00	0.00	0.00	0.00	0.00	0.00	0.00	0.00	0.00	0.00	0.00
206	0.00	0.00	0.00	0.00	0.00	0.00	0.00	0.00	0.00	0.00	0.00	0.00	0.00	0.00	0.00	0.00	0.00	0.00	0.00	0.00
208	0.00	0.00	0.00	0.00	0.00	0.00	0.00	0.00	0.00	0.00	0.00	0.00	0.00	0.00	0.00	0.00	0.00	0.00	0.00	0.00
210	0.00	0.00	0.00	0.00	0.00	0.00	0.00	0.00	0.00	0.00	0.00	0.00	0.00	0.00	0.00	0.00	0.00	0.00	0.00	0.00
212	0.00	0.00	0.00	0.00	0.00	0.00	0.00	0.00	0.00	0.00	0.00	0.00	0.00	0.00	0.00	0.00	0.00	0.00	0.00	0.00
215	0.00	0.00	0.00	0.00	0.00	0.00	0.00	0.00	0.00	0.00	0.00	0.00	0.00	0.00	0.00	0.00	0.00	0.00	0.00	0.00
217	0.00	0.00	0.00	0.00	0.00	0.00	0.00	0.00	0.00	0.00	0.00	0.00	0.00	0.00	0.00	0.00	0.00	0.00	0.00	0.00
219	0.00	0.00	0.00	0.00	0.00	0.00	0.00	0.00	0.00	0.00	0.00	0.00	0.00	0.00	0.00	0.00	0.00	0.00	0.00	0.00
221	0.00	0.00	0.00	0.00	0.00	0.00	0.00	0.00	0.00	0.00	0.00	0.00	0.00	0.00	0.00	0.00	0.00	0.00	0.00	0.00
222	0.00	0.00	0.00	0.00	0.00	0.00	0.00	0.00	0.00	0.00	0.00	0.00	0.00	0.00	0.00	0.00	0.00	0.00	0.00	0.00
224	0.00	0.00	0.00	0.00	0.00	0.00	0.00	0.01	0.00	0.00	0.00	0.00	0.00	0.00	0.00	0.00	0.00	0.00	0.00	0.00
225	0.00	0.00	0.00	0.00	0.00	0.01	0.00	0.00	0.00	0.00	0.00	0.00	0.00	0.00	0.00	0.01	0.00	0.01	0.00	0.01
226	0.01	0.00	0.00	0.00	0.00	0.00	0.00	0.00	0.00	0.00	0.00	0.00	0.00	0.00	0.00	0.00	0.00	0.00	0.00	0.00
227	0.01	0.00	0.01	0.00	0.01	0.01	0.01	0.01	0.00	0.00	0.00	0.00	0.00	0.00	0.01	0.01	0.00	0.01	0.00	0.00
228	0.00	0.00	0.00	0.00	0.00	0.00	0.00	0.00	0.04	0.00	0.00	0.00	0.00	0.00	0.00	0.00	0.00	0.00	0.00	0.00
229	0.04	0.00	0.05	0.04	0.00	0.00	0.00	0.00	0.00	0.04	0.00	0.00	0.04	0.04	0.05	0.00	0.00	0.00	0.03	0.04
230	0.08	0.15	0.08	0.00	0.12	0.09	0.12	0.16	0.00	0.00	0.10	0.10	0.07	0.00	0.09	0.09	0.08	0.16	0.06	0.07
231	0.00	0.00	0.00	0.00	0.00	0.00	0.00	0.00	0.00	0.00	0.00	0.00	0.00	0.00	0.00	0.00	0.00	0.00	0.00	0.00
232	0.16	0.16	0.20	0.22	0.19	0.17	0.21	0.17	0.23	0.25	0.18	0.18	0.21	0.00	0.15	0.20	0.22	0.22	0.26	0.22
233	0.00	0.00	0.00	0.00	0.00	0.00	0.00	0.00	0.00	0.00	0.00	0.00	0.00	0.18	0.00	0.00	0.00	0.00	0.00	0.00
234	0.06	0.06	0.05	0.00	0.05	0.06	0.06	0.06	0.00	0.00	0.08	0.06	0.00	0.00	0.00	0.08	0.07	0.08	0.05	0.00
235	0.00	0.00	0.00	0.00	0.00	0.00	0.00	0.00	0.00	0.00	0.00	0.00	0.00	0.00	0.00	0.00	0.00	0.00	0.00	0.00
236	0.00	0.03	0.02	0.00	0.00	0.00	0.00	0.03	0.00	0.00	0.00	0.00	0.00	0.00	0.00	0.04	0.03	0.00	0.00	0.00
237	0.04	0.00	0.00	0.00	0.01	0.04	0.04	0.00	0.01	0.00	0.04	0.04	0.00	0.00	0.00	0.00	0.00	0.02	0.01	0.00
238	0.00	0.00	0.00	0.00	0.00	0.00	0.00	0.00	0.00	0.00	0.00	0.00	0.00	0.00	0.00	0.00	0.00	0.00	0.00	0.00
241	0.00	0.00	0.02	0.00	0.00	0.00	0.00	0.00	0.00	0.06	0.00	0.00	0.00	0.00	0.00	0.00	0.00	0.00	0.00	0.00
242	0.03	0.00	0.00	0.00	0.03	0.03	0.03	0.02	0.01	0.00	0.02	0.00	0.01	0.00	0.02	0.01	0.02	0.00	0.00	0.03
243	0.00	0.00	0.00	0.00	0.00	0.00	0.00	0.00	0.00	0.00	0.00	0.00	0.00	0.00	0.00	0.00	0.00	0.00	0.00	0.00
244	0.00	0.00	0.01	0.00	0.00	0.00	0.00	0.00	0.00	0.00	0.00	0.00	0.01	0.00	0.00	0.01	0.00	0.00	0.00	0.00
245	0.01	0.00	0.00	0.00	0.00	0.01	0.00	0.00	0.00	0.00	0.00	0.00	0.00	0.00	0.00	0.00	0.00	0.00	0.00	0.00
246	0.00	0.00	0.01	0.01	0.00	0.00	0.00	0.01	0.00	0.00	0.00	0.00	0.01	0.01	0.02	0.00	0.00	0.00	0.00	0.01
247	0.00	0.00	0.00	0.00	0.00	0.00	0.00	0.00	0.00	0.00	0.00	0.00	0.00	0.00	0.00	0.00	0.00	0.00	0.00	0.00
248	0.01	0.00	0.01	0.01	0.00	0.01	0.00	0.01	0.02	0.00	0.00	0.00	0.00	0.02	0.01	0.00	0.00	0.00	0.01	0.01
249	0.00	0.00	0.00	0.00	0.00	0.00	0.00	0.00	0.00	0.00	0.00	0.00	0.00	0.00	0.00	0.00	0.00	0.00	0.00	0.00
250	0.00	0.00	0.00	0.00	0.01	0.00	0.00	0.00	0.00	0.00	0.00	0.00	0.00	0.00	0.00	0.00	0.00	0.00	0.00	0.01
251	0.00	0.00	0.00	0.00	0.00	0.00	0.00	0.00	0.00	0.00	0.00	0.00	0.00	0.00	0.00	0.00	0.00	0.00	0.00	0.00
255	0.00	0.00	0.00	0.00	0.00	0.00	0.00	0.00	0.00	0.00	0.00	0.00	0.00	0.00	0.00	0.00	0.00	0.00	0.00	0.00
261	0.00	0.00	0.01	0.01	0.00	0.00	0.00	0.00	0.01	0.00	0.00	0.00	0.01	0.01	0.01	0.00	0.00	0.00	0.00	0.01
264	0.00	0.00	0.00	0.00	0.00	0.00	0.00	0.00	0.00	0.00	0.00	0.00	0.00	0.00	0.00	0.00	0.00	0.00	0.00	0.00
265	0.02	0.00	0.00	0.00	0.00	0.00	0.00	0.00	0.00	0.00	0.00	0.00	0.00	0.00	0.00	0.00	0.00	0.00	0.00	0.00
266	0.00	0.00	0.02	0.03	0.02	0.02	0.02	0.01	0.02	0.03	0.00	0.00	0.02	0.02	0.02	0.01	0.02	0.01	0.02	0.03
271	0.00	0.00	0.00	0.00	0.00	0.00	0.00	0.00	0.00	0.00	0.00	0.00	0.00	0.00	0.00	0.00	0.00	0.00	0.00	0.00
273	0.00	0.00	0.00	0.00	0.00	0.00	0.00	0.00	0.00	0.00	0.00	0.00	0.00	0.00	0.00	0.00	0.00	0.00	0.00	0.00
275	0.00	0.00	0.00	0.00	0.00	0.00	0.00	0.00	0.00	0.00	0.00	0.00	0.00	0.00	0.00	0.00	0.00	0.00	0.00	0.00
278	0.00	0.00	0.00	0.01	0.00	0.00	0.00	0.00	0.01	0.00	0.00	0.00	0.00	0.00	0.00	0.00	0.00	0.00	0.00	0.00
279	0.00	0.00	0.00	0.00	0.00	0.00	0.00	0.00	0.00	0.00	0.00	0.00	0.00	0.00	0.00	0.00	0.00	0.00	0.00	0.00
283	0.01	0.00	0.01	0.00	0.00	0.01	0.01	0.02	0.00	0.00	0.00	0.00	0.00	0.00	0.01	0.01	0.00	0.01	0.00	0.01
284	0.00	0.00	0.00	0.00	0.00	0.00	0.00	0.00	0.00	0.00	0.00	0.00	0.00	0.00	0.00	0.01	0.00	0.00	0.00	0.00
285	0.00	0.00	0.01	0.01	0.00	0.00	0.00	0.01	0.01	0.00	0.00	0.00	0.00	0.01	0.01	0.01	0.00	0.01	0.00	0.01
288	0.00	0.00	0.00	0.00	0.00	0.00	0.00	0.00	0.00	0.00	0.00	0.00	0.00	0.00	0.00	0.00	0.00	0.00	0.00	0.00
294	0.00	0.00	0.00	0.00	0.00	0.00	0.00	0.00	0.00	0.00	0.00	0.00	0.00	0.00	0.00	0.00	0.00	0.00	0.00	0.00
298	0.00	0.00	0.00	0.00	0.00	0.00	0.00	0.00	0.00	0.00	0.00	0.00	0.00	0.00	0.01	0.00	0.00	0.00	0.00	0.00
301	0.00	0.00	0.00	0.01	0.01	0.00	0.00	0.00	0.00	0.00	0.00	0.00	0.00	0.01	0.01	0.00	0.00	0.00	0.00	0.01
305	0.03	0.00	0.00	0.00	0.00	0.00	0.00	0.00	0.00	0.00	0.00	0.00	0.00	0.00	0.00	0.00	0.00	0.00	0.00	0.00
306	0.00	0.00	0.04	0.06	0.03	0.02	0.02	0.02	0.04	0.05	0.03	0.02	0.04	0.05	0.06	0.03	0.02	0.02	0.03	0.06
307	0.00	0.00	0.00	0.03	0.00	0.00	0.00	0.00	0.00	0.00	0.00	0.00	0.00	0.00	0.00	0.00	0.00	0.00	0.00	0.00
308	0.00	0.00	0.02	0.00	0.03	0.00	0.02	0.01	0.06	0.00	0.00	0.00	0.02	0.04	0.00	0.00	0.00	0.00	0.02	0.00
309	0.05	0.00	0.03	0.05	0.07	0.05	0.03	0.00	0.09	0.09	0.03	0.00	0.00	0.08	0.03	0.05	0.00	0.00	0.05	0.02
310	0.00	0.00	0.00	0.00	0.00	0.00	0.00	0.03	0.00	0.00	0.00	0.02	0.04	0.00	0.00	0.00	0.03	0.01	0.00	0.00
313	0.00	0.00	0.00	0.00	0.00	0.00	0.00	0.00	0.00	0.00	0.00	0.00	0.00	0.00	0.00	0.00	0.00	0.00	0.00	0.00
314	0.00	0.00	0.00	0.00	0.00	0.00	0.00	0.00	0.00	0.00	0.00	0.00	0.00	0.00	0.00	0.00	0.00	0.00	0.00	0.00
316	0.00	0.00	0.01	0.00	0.00	0.00	0.00	0.00	0.00	0.02	0.00	0.00	0.00	0.00	0.01	0.00	0.00	0.00	0.00	0.01
317	0.00	0.00	0.00	0.00	0.00	0.00	0.00	0.00	0.00	0.00	0.00	0.00	0.00	0.00	0.00	0.00	0.00	0.00	0.00	0.00
319	0.00	0.00	0.00	0.00	0.00	0.00	0.00	0.00	0.00	0.00	0.00	0.00	0.00	0.00	0.00	0.00	0.00	0.00	0.00	0.00
321	0.00	0.00	0.00	0.00	0.00	0.00	0.00	0.00	0.00	0.00	0.00	0.00	0.00	0.00	0.00	0.00	0.00	0.00	0.00	0.00
322	0.00	0.00	0.00	0.00	0.00	0.00	0.00	0.00	0.00	0.00	0.00	0.00	0.00	0.00	0.01	0.00	0.00	0.00	0.00	0.01
323	0.00	0.00	0.00	0.00	0.00	0.00	0.00	0.00	0.01	0.00	0.00	0.00	0.00	0.00	0.00	0.00	0.00	0.00	0.00	0.00
324	0.00	0.00	0.00	0.00	0.00	0.00	0.00	0.00	0.00	0.00	0.00	0.00	0.00	0.00	0.00	0.00	0.00	0.00	0.00	0.01
325	0.00	0.00	0.00	0.00	0.00	0.00	0.00	0.00	0.00	0.00	0.00	0.00	0.00	0.00	0.00	0.00	0.00	0.00	0.00	0.00
328	0.00	0.00	0.00	0.00	0.00	0.00	0.00	0.00	0.00	0.00	0.00	0.00	0.00	0.00	0.00	0.00	0.00	0.00	0.00	0.00
329	0.00	0.00	0.01	0.01	0.00	0.00	0.00	0.00	0.00	0.00	0.00	0.00	0.00	0.01	0.01	0.01	0.00	0.01	0.00	0.01
330	0.00	0.00	0.00	0.00	0.00	0.00	0.00	0.00	0.00	0.00	0.00	0.00	0.00	0.00	0.00	0.00	0.00	0.00	0.00	0.00
331	0.00	0.00	0.00	0.00	0.00	0.00	0.00	0.00	0.00	0.00	0.00	0.00	0.00	0.00	0.00	0.00	0.00	0.00	0.00	0.00
345	0.00	0.00	0.00	0.00	0.00	0.00	0.00	0.01	0.00	0.00	0.00	0.00	0.00	0.00	0.00	0.00	0.00	0.00	0.00	0.00
373	0.00	0.19	0.00	0.00	0.00	0.00	0.00	0.00	0.00	0.00	0.00	0.00	0.00	0.00	0.00	0.00	0.00	0.00	0.00	0.00

© 2013 by the authors; licensee MDPI, Basel, Switzerland. This article is an open access article distributed under the terms and conditions of the Creative Commons Attribution license (http://creativecommons.org/licenses/by/3.0/).
